# Beyond the IFA: Revisiting the ELISA as a More Sensitive, Objective, and Quantitative Evaluation of Spotted Fever Group *Rickettsia* Exposure

**DOI:** 10.3390/pathogens10020088

**Published:** 2021-01-20

**Authors:** Navatha Alugubelly, John V. Stokes, Claire E. Cross, Anne-Marie L. Ross, Anna E. Crawford, Gabrielle F. Fiihr, Andrea S. Varela-Stokes

**Affiliations:** Department of Comparative Biomedical Sciences, College of Veterinary Medicine, Mississippi State University, Mississippi State, MS 39762, USA; nalugubelly@cvm.msstate.edu (N.A.); jstokes@cvm.msstate.edu (J.V.S.); cec883@msstate.edu (C.E.C.); ar2324@msstate.edu (A.-M.L.R.); aec23@uab.edu (A.E.C.); gff22@msstate.edu (G.F.F.)

**Keywords:** *Rickettsia parkeri*, *Rickettsia amblyommatis*, guinea pig, serology, tick-borne diseases, *Amblyomma*, rickettsial diseases

## Abstract

Based on limited serological studies, at least 10% of the US population has been exposed to spotted fever group *Rickettsia* (SFGR) species. The immunofluorescence antibody assay (IFA) has been the gold standard for the serodiagnosis of rickettsial infections such as spotted fever rickettsiosis (SFR). However, the IFA is semi-quantitative and subjective, requiring a high level of expertise to interpret it correctly. Here, we developed an enzyme-linked immunosorbent assay (ELISA) for the serodiagnosis of *Rickettsia parkeri* infection in the guinea pig. Our ELISA is an objective, quantitative, and high-throughput assay that shows greater sensitivity and resolution in observed titers than the IFA. We methodically optimized relevant parameters in sequence for optimal signal-to-noise ratio and low coefficient of variation% values. We used a guinea pig model as it is a part of our overall research efforts to understand the immunological and clinical response to SFGR species after tick transmission. Guinea pigs are a useful model to study SFR and show clinical signs of SFR, such as fever and eschars. We anticipate that this assay will be easily adapted to other hosts, including humans and other SFGR species.

## 1. Introduction

Rickettsiae are obligate intracellular, Gram-negative bacteria associated with various arthropod vectors such as ticks, fleas, and mites [1,2]. Spotted fever rickettsiosis (SFR) is a zoonotic tick-borne disease caused by pathogenic spotted fever group *Rickettsia* (SFGR) species. There are more than 20 known SFGR species worldwide. *Rickettsia rickettsii,* the agent of Rocky Mountain spotted fever (RMSF), is the most virulent SFGR species in North America and was long considered the only pathogenic SFGR species in the US. This belief changed in 2004 when the index case of a rickettsiosis caused by *Rickettsia parkeri* was described in a patient from VA, USA [3]. *Rickettsia parkeri* is an emerging pathogenic SFGR species and agent of SFR; it is primarily transmitted via *Amblyomma maculatum* (Gulf Coast tick) in the southern USA [4]. A closely related tick species in the *A. maculatum* group, *Amblyomma triste*, was also recently identified as the vector for *R. parkeri* in SFR cases from Arizona [5]. In 2010, *Rickettsia* 364D became the third known tick-borne pathogenic SFGR species in the US; it is transmitted to humans through *Dermacentor occidentalis* along the West Coast [6]. Additionally, there are presumably non-pathogenic species in the SFGR, most notably *R. amblyommatis*, which is associated with *Amblyomma americanum* (lone star tick) [7,8].

Seroepidemiologic studies consistently show exposure to SFGR species among a significant percentage of the US population. The largest serological survey of rickettsial infection in children living in the southeastern and south-central US found that 12% of children tested were seropositive; that is, they had rickettsial antibody titers of at least 64 [9]. Another seroepidemiologic study in North Carolina identified antibodies to one or more SFGR species, including *R. parkeri*, *R. amblyommatis*, and *R. rickettsii*, in 21 of 106 patients tested [10]. A seroprevalence study of SFGR species and *Anaplasma phagocytophilum*, the agent of human granulocytic anaplasmosis, demonstrated seropositivity of 6% and 2.6% in tested persons, respectively [11]. In a study of human exposure to four SFGR, *R. rickettsii*, *R. parkeri*, *R. amblyommatis*, and *R. montanensis*, all samples tested were cross-reactive to at least two SFGR species, with a minimum antibody titer of 64 [12]. Based on published seroprevalence data to date, humans are at risk of exposure to more than one agent of SFR and can develop antibodies to both pathogenic and non-pathogenic SFGR species.

Guinea pigs have been invaluable for identifying rickettsiae and understanding tick-borne rickettsial diseases since the early 20th century [13,14]. However, beginning in the late 1980s, the guinea pig was mostly replaced by the mouse model for SFR and other infectious diseases. This new preference in models was primarily driven by the widespread availability of genetically modified murine models and mouse-specific reagents [15]. Despite this shift, guinea pigs remain a more suitable laboratory animal for translational studies because their immune system more closely models that of a human than a mouse’s immune system does. Moreover, guinea pigs have a larger blood volume than mice, making them more conducive to repetitive sampling in a longitudinal study, and are generally easy to handle and practical for tick transmission studies [16,17,18]. As we utilize a guinea pig–*Amblyomma–Rickettsia* system, correctly monitoring exposure to tick-transmitted rickettsiae in guinea pigs is critical for understanding the immunological and clinical response to SFGR.

Clinical symptoms of SFR in humans are flu-like, including fever and headache, and often include a characteristic rash or, in the case of *R. parkeri* and *Rickettsia* 364D, an eschar at the site of the tick bite [6,19]. Symptoms may mimic other illnesses, especially in the absence of dermal lesions or a history of tick bite. Specific detection of SFGR in clinical specimens (e.g., biopsies) by PCR or immunohistochemistry is not always possible and can be challenging. While confirmation of SFR can be made by detecting rickettsial DNA or antigen, or by culture isolation of rickettsiae, the presence of a four-fold increase in antibodies in paired serum samples is also sufficient to diagnose SFR [20]. However, one should note that serodiagnosis of SFR is not as useful in clinical decision-making because detectable antibodies appear late relative to disease development and the need for rapid treatment. Currently, the immunofluorescence antibody assay (IFA) is considered the gold standard for serodiagnosis of SFR. However, the IFA may be subjective, requiring a well-trained professional to interpret the results appropriately, and remains a semi-quantitative, low-throughput diagnostic assay. To overcome the shortcomings of the IFA, we developed an enzyme-linked immunosorbent assay (ELISA) for SFR diagnosis as the ELISA is objective, quantitative, has higher throughput, and is more sensitive compared to the IFA. As serologic assays are more useful for epidemiologic studies, an improved assay remains critical to assess population exposure and make informed public health decisions.

## 2. Results and Discussion

Here, we addressed the need for an improved method of identifying SFGR exposure using the guinea pig model for SFR. Through methodically optimizing each parameter in sequence, we developed an ELISA for detecting *R. parkeri* exposure with a higher level of sensitivity and confidence of positivity than is possible with an IFA. First, through an initial titration, we determined the secondary antibody concentration that would produce an acceptable signal-to-noise ratio (S/N). Other parameters were subsequently optimized, after which a final optimization of the secondary antibody became feasible. We performed the initial secondary antibody titration by preparing a serial dilution of 1:500 to 1:32,000 on a plate with *R. parkeri* antigen only and selecting the dilution where an inflection point on the curve of optical density (OD) versus dilution occurred. This value was 1:8000; we used this dilution to optimize other parameters and then determined the final, optimal dilution based on those conditions (Figure S1). We selected the blocking agent based on the greatest reduction of background noise. We compared ELISA Ultrablock (Bio-Rad, Hercules, CA, USA), ELISA BSA Block (Bio-Rad), and SuperBlock™ (Thermo Scientific, Waltham, MA, USA). Ultrablock suppressed background noise most effectively and was thus chosen for the assay. Interestingly, Ultrablock is formulated using a fish extract and is, therefore, more phylogenetically distinct from guinea pig as compared to BSA; SuperBlock™, which contains a “proprietary non-relevant protein”, also outperformed BSA as a blocking agent.

Next, we tested ELISA plates of varying hydrophobicity, including hydrophilic (Thermo MaxiSorp, Waltham, MA, USA), slightly hydrophilic (Thermo MediSorp, Waltham, MA, USA), and hydrophobic (Thermo PolySorp, Waltham, MA, USA) plates. Very hydrophilic plates (Thermo MultiSorp, Waltham, MA, USA) were not tested. Our rationale for not testing the very hydrophilic plates was that since the best combination of CV% [coefficient of variation% = (standard deviation/mean) × 100] and S/N was produced by the slightly hydrophilic plates, with a marginally better CV% on the hydrophobic plate and somewhat better S/N on the hydrophilic plate, it seemed unlikely that a very hydrophilic plate would be superior in both respects. Then, we assessed different *R. parkeri* antigen densities, including 1 × 10^7^, 2 × 10^7^, 4 × 10^7^, 5 × 10^7^, 6 × 10^7^, and 8 × 10^7^ rickettsiae per well. Since CV% was inversely correlated to the antigen concentration, we chose 5 × 10^7^ rickettsiae per well as this was the lowest antigen concentration that produced consistent CV% values less than 5. Finally, we refined the titration of the secondary antibody concentration by reducing it from 1:8000 to 1:12,000, the lowest concentration without apparent signal loss.

Plasma from a guinea pig hyperimmunized with *R. parkeri* was used to generate the standard curve. After determining end-point titer using an IFA and plasma diluted to a finer resolution than a standard two-fold dilution, we then made stock dilutions of plasma at a 1200 titer. When the standard curve was generated, the stock was further diluted to 1:7000. We did this so the ELISA plate incubation for detection could proceed for 30 min with the final OD of the 1200-titer standard being consistently ≤3. We believed this was optimal because a 30-min TMB substrate incubation time would allow for the inevitable small differences in timing while conducting the assay without significantly affecting plate-to-plate reproducibility, and 3 OD does not approach the 4 OD high-end range of the plate reader. For assessing the assay’s reproducibility and sensitivity, our “seropositive” control plasma samples came from the 1200-titer stock diluted to 1:14,000 so the calculated titer would fall in the middle of the standard curve. The negative controls and the unknowns which we tested were diluted to 1:1200 so that the samples would have the same level of background as the 1200-titer standard. This method of diluting negative controls and unknowns ensured that their background signal was always higher than the background signal of the 75-titer standard, which was diluted to 1:112,000, thus further reducing the probability of a false positive.

Since positivity was defined as when the mean ($\bar{x}$) OD of the unknown was > $\bar{x}$ OD + 3 SD (standard deviation) of the negative control, and the negative controls and unknowns had more background than the 75-titer standard, it seemed unlikely that a false positive would occur under these circumstances. Further, after optimization, the 75-titer OD was greater than the $\bar{x}$ OD of the negative + 5 SD in all cases, suggesting a less than 1 in 3.5 million chance of a false positive of an unknown whose titer falls within the standard curve. This level of sensitivity also suggests that a simple non-quantitative determination of positivity can be made below the standard curve with high confidence by applying the $\bar{x}$ OD + 3 SD of the negative control rule.

Following optimization, we performed the assay three times on three different days using plates from the same lot to assess intra- and inter-plate reproducibility (Tables S2–S5). Before commencing development, we set the following goals for plate performance: intra-plate CV% ≤ 5; inter-plate CV% ≤ 10; standard curve R^2^ ≥ 0.985; and deviation of the highest and lowest single OD values ≤ 10% from the mean OD. We believed these were ambitious goals for an in-house whole-cell ELISA and were pleased with the results. For intra-plate reproducibility, CV% values were ≤ 5 for all samples except for a single “no plasma”, which had a CV% of 6.2 in plate 3. The deviation of the highest and lowest single OD values from the mean was ≤ 10% for all samples except the “positive control” and “no plasma” samples also in plate 3, which had values of 10.2 and 11.3, respectively. The R^2^ = 1 for all three plates. Inter-plate CV% was ≤ 10 for all the samples except three of the standards, 1200, 600, and 150, which had values of 10.5, 12.8, and 10.2, respectively.

**Figure 1:**
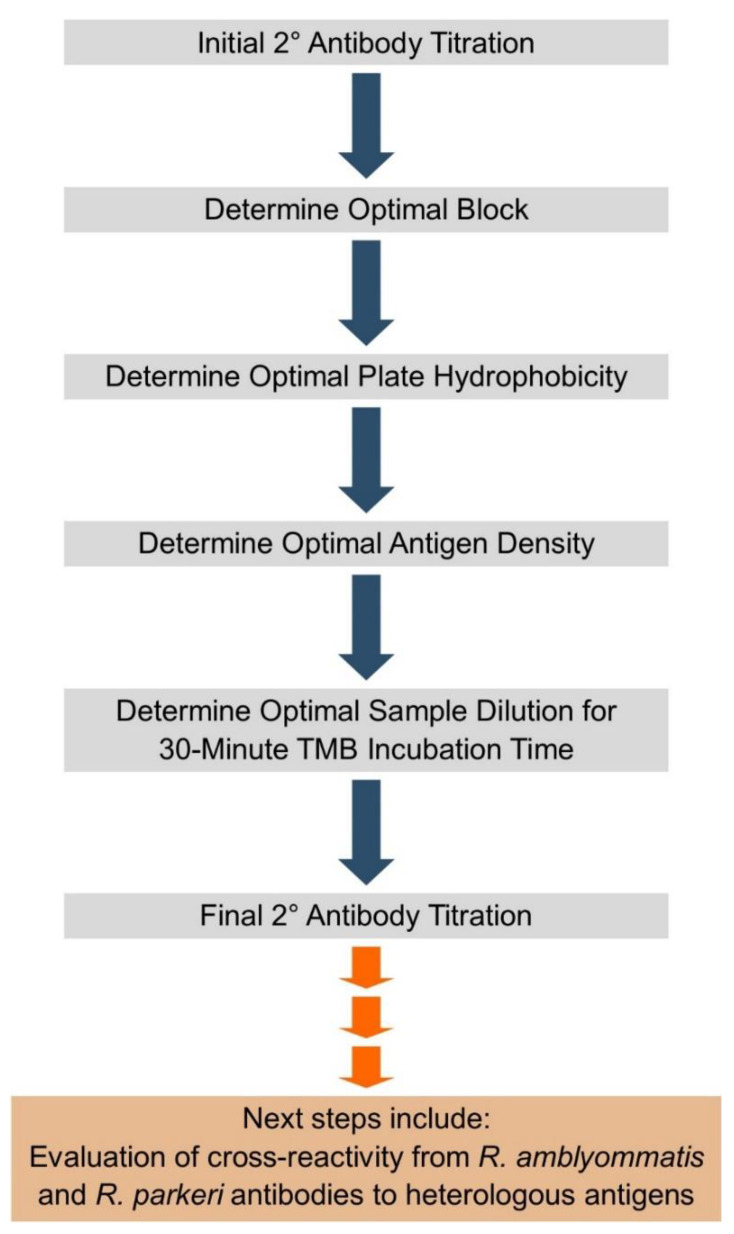
Schematic of process for developing optimized ELISA for serodiagnosis of SFR (spotted fever rickettsiosis) using guinea pig model, including next step for assay.

To directly compare ELISA to IFA results, we evaluated “unknown” ELISA samples with titers previously determined by an IFA and read by an experienced researcher who has performed them for nearly 20 years (A.S.V-S.). One sample with an IFA titer of 64 had a titer of 107 in the ELISA (expected between 64 and 127); another sample with an IFA titer of 512 had a titer of 955 on the ELISA (expected between 512 and 1023). These results suggest that the data generated by the ELISA are consistent with, but more quantitative than, those generated by the IFA. We also analyzed plasma samples from guinea pigs exposed to *R. amblyommatis*-infected *A. americanum* ticks. These samples were positive (>$\bar{x}$ OD + 3 SD of the negative control) but outside the range of the standard curve. These results suggest that there is some cross-reactivity of *R. amblyommatis* antibody to *R. parkeri* antigen. In the future, we will incorporate additional replicates of pre- and post-exposure samples from SFGR experimentally infected guinea pigs as unknowns as we progress in further validating the assay. Additionally, we plan a more comprehensive assessment of cross-reactivity by preparing different plates with *R. parkeri* and *R. amblyommatis* antigen and assessing cross-reactivity in both directions (Figure 1). By concurrently running ELISA assays plated with antigen from different *Rickettsia* spp., we could in principle differentiate pathogenic and non-pathogenic SFGR, much like what is sometimes done using the IFA, but with the ELISA having greater differential resolution. The *Rickettsia* sp. antigen used for additional cross-reactivity studies would be expanded beyond *R. parkeri* and *R. amblyommatis* to include other SFGR such as *R. rickettsii*, *Rickettsia* 364D, and *R. montanensis*.

This assay has two principal limitations. The first is that it was developed using guinea pig plasma from our current guinea pig–*Amblyomma–Rickettsia* system; however, human plasma or serum should generate similar results by adapting the assay for human samples. Second, the standard curve was constructed based on the IFA titer value; however, the IFA titer was determined by performing dilutions to a finer resolution (5000, 5200, 5400, etc.) than the more typical serial dilutions (3200, 6400, 12,800, etc.). Aside from these limitations, the ELISA is a simple and convenient way to detect SFGR exposure. The assay uses a single serum dilution compared to IFA, which uses a series of dilutions. Previously, the ELISA was tested for the serodiagnosis of SFR such as RMSF [21], Mediterranean spotted fever [22], and other rickettsial diseases such as murine typhus [23] and scrub typhus [24]. The limitation of those ELISA techniques included reporting seropositivity in OD and using arbitrary cutoffs to determine whether a sample was seropositive or seronegative. These limitations make the results less intuitive to clinicians, and those techniques were not developed further as diagnostic tools. In our method, we have converted the OD to the more familiar titer using a standard curve. Moreover, our assay does not rely on arbitrary cutoffs to determine seropositivity but is calculated directly from the negative control and based on the statistical probability of a false positive being <0.15%; that is, positivity is defined as >$\bar{x}$ OD + 3 SD of the negative controls.

## 3. Materials and Methods

### 3.1. Sample Collection

Whole blood was collected via jugular venipuncture from isoflurane-anesthetized male Hartley guinea pigs (Charles River Laboratories) ranging in age from 3 to ~29 months of age. Blood (250–450 µL) was transferred to individual EDTA microcontainers and centrifuged at 3000× *g* to separate plasma. Plasma was divided into multiple aliquots to minimize freeze–thaw cycles. All experimental animal work was performed under protocols (No. 18-267 and No. 17-166, with renewal No. 20-210) approved by the Institutional Animal Care and Use Committee (IACUC) at Mississippi State University.

Seronegative control plasma was collected from a guinea pig that was not previously exposed to *Rickettsia* spp. or subjected to tick-feeding. Early in the optimization process, pre-exposure seronegative control plasma was available from the same guinea pig from which we used the post-exposure seropositive control plasma for the entire optimization procedure and final reproducibility assessment. As we progressed in the assay development, however, we required additional seronegative plasma and collected this from a different guinea pig. Seropositive control plasma was collected from a guinea pig that was injected subcutaneously with 0.2 mL of ISE6 cells co-cultivated with *R. parkeri* (Portsmouth, passage 9 or 11). *R. parkeri* co-cultures were maintained in L15B300 medium [25] supplemented with 20% fetal bovine serum (FBS) and 10% tryptose phosphate broth (TBP). *Rickettsia parkeri* co-cultured in ISE6 cells was grown in non-vented tissue culture flasks at 32 °C in an incubator without supplemental CO_2_. Plasma collected from the *R. parkeri*-injected guinea pig on day post-injection (DPI) 21 had a titer of 256, determined by immunofluorescence antibody assay (IFA) using antigen-coated slides made in-house with *R. parkeri* (Portsmouth) co-cultivated in Vero cells. To boost the titer for use as a positive control, we challenged the guinea pig on DPI-21 using 10^5^ *R. parkeri*-infected *Ixodes scapularis* embryonic (ISE6) tick cells. Plasma collected on DPI 59 had an IFA titer ≈ 7000, determined after subjecting plasma to two-fold dilutions, starting with a 1:64 dilution for an end-point titer of 6400 and further resolving this titer with dilutions between 1:5000 and 1:15,000 (1:5000, 1:5200, 1:5400, etc.) for an end-point titer ≈ 7000.

Guinea pig plasma samples used for “unknowns” included plasma from guinea pigs exposed to *R. parkeri* via transmission by infected *A. maculatum* (confirmed positive for *R. parkeri* by qPCR testing of single tick leg and hemolymph). In addition, we tested plasma from two guinea pigs exposed to *R. amblyommatis*-infected *A. americanum*, 21 days post-tick-feeding. For a complete list of resources, see Table S1.

### 3.2. Antigen Preparation and Plating

We chose whole bacterial cell antigen and enriching bacteria through tabletop centrifugation as this was a simple method accessible to most laboratories, eliminating the need for a superspeed centrifuge for gradient purification or other equipment for bacterial cell lysis (e.g., French press). Further, cell lysis would add an unnecessary step considering that antibody responses are typically against cell surface antigens. Our goal was to develop a simple approach practical for most laboratories.

*Rickettsia parkeri* (Portsmouth; passage 11 or 12) was co-cultivated in Vero cell culture to prepare antigen using T75 vented flasks and MEM supplemented with 10% FBS. *Rickettsia parkeri* co-cultured in Vero cells was grown in vented tissue culture flasks at 37 °C in an incubator with 5% CO_2_. Briefly, when gross cytopathic effect was evident, we harvested infected cells by scraping the cells using a cell scraper and blowing loosely adherent cells off using a serological pipet. We transferred culture suspensions to a 50- or 60-mL syringe equipped with a 25-g needle from which the plunger had been removed and the syringe situated on top of a 50-mL tube. The plunger was replaced, and the suspension passed through the needle to break apart cell clumps. The suspension was subsequently passed through a 27-g needle to mechanically rupture some cells to release intracellular rickettsiae. After centrifuging the suspension at 1000× *g* for 10 min to pellet most cellular debris, the supernatant was passed through a Whatman 3-µm filter to further clean the suspension and collect rickettsiae, then centrifuged at 12,000× *g* for 10 min to pellet rickettsiae. Pellets were washed three times by resuspending in sterile PBS (pH 7.4) and centrifuging at 12,000× *g* for 10 min each time.

A 1:100 dilution of the final suspension of bacteria was stained using Live/Dead^™^ BacLight™ Bacterial Viability Kit, with the stained bacteria further diluted to a final 1:1000 dilution for counting using a Petroff–Hauser counting chamber. The remainder of the suspension was heat-killed for one hour in a 56 °C water bath to be used for plating. The total number of bacteria needed to seed wells was centrifuged (12,000× *g*) for 10 min and resuspended in 1× coating buffer, with 100 µL dispensed into each well of the ELISA strip using a multichannel pipet. The plate with strips was sealed using sealing tape, covered in foil to protect from light, and placed at 4 °C overnight (~24 h) to allow the antigen to adhere to well bottoms. After incubation, the coating buffer was dumped by flicking plates and tapping on absorbent paper to remove residual liquid. We used a plate washer to wash strips two times with 300 µL/well 1× wash buffer, quickly visualized antigen density on wells under an inverted microscope without allowing wells to dry, and then blocked plates overnight at RT using 200 µL/well Ultrablock. Strips were covered using sealing tape and then foil to protect from light while blocking. Ultrablock was then removed by flicking the plate and then tapping on absorbent paper. Finally, wells were dried under a biosafety cabinet with the fan on and light off for 3 h, then placed in a sealed bag or drawer with desiccant and kept at 4 °C until use. For a complete list of resources, see Table S1.

### 3.3. Assay

The whole-cell *R. parkeri*-coated ELISA plate and other required reagents were brought to room temperature an hour before the start of the assay. The seropositive control plasma (titer 7000) was diluted 1:5.83 with FCM-PBS (1% bovine serum albumin in PBS filtered to 0.1 μm) to adjust the titer from 7000 to 1200, and this diluted plasma was termed stock control plasma and was used for the standard curve. The leftover stock plasma was stored at −20 °C in 12-µL aliquots in protein LoBind tubes for use in future experiments. The stock plasma was further diluted at a 1:1200 dilution to yield a final dilution of 1:7000, which was used as the highest standard curve point and defined as titer 1200. The plasma with 1:7000 dilution was serially diluted until 1:112,000 (titer = 75) through two-fold dilutions. The seropositive control was used at a 1:14,000 dilution, whereas the seronegative control was used at a 1:1200 dilution to match directly with the dilution of the highest standard curve point (standard 1200). Previously prepared 1:5.83 dilution samples (archives) were also included in the assay to assess these samples’ stability over time. Archives were used at a 1:7000 dilution. “No plasma” wells had only secondary antibody added. All of the dilutions were prepared using FCM-PBS.

**Figure 2:**
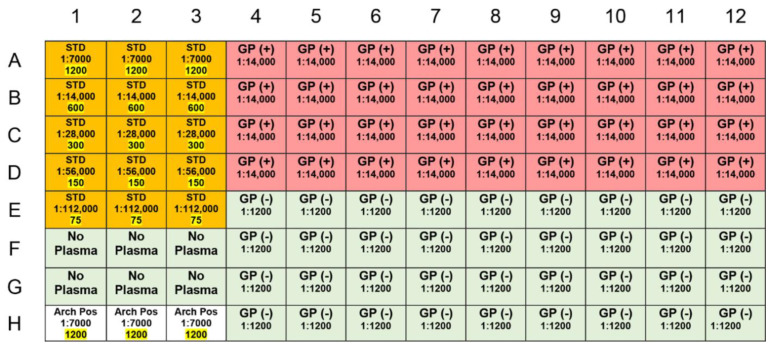
Plate set-up displaying the location and number of wells assigned to different sample types including positive control [GP (+) 1:14,000], negative control [GP (−) 1:1200], archives (arch pos 1:7000), no plasma, and standards (STD 1200 to STD 75).

Appropriately diluted plasma (100 µL) was added to corresponding wells according to the plate setup in Figure 2. FCM-PBS (100 µL) was added to “no plasma” samples. The plate was covered with sealing tape and incubated at room temperature for 2 h with shaking at 250 rpm. After 2 h, the plate was washed four times with 300 µL 1× wash buffer using a plate washer. The plate was soaked for 5 min before discarding the buffer during the fourth wash. HRP-conjugated secondary antibody (100 µL at a 1:12,000 dilution in FCM-PBS) was added to each well. The plate was covered with sealing tape and incubated at room temperature for 1 h with shaking at 250 rpm. The plate was washed four times again as above. After adding 100 µL of TMB substrate to each well, the plate was covered with aluminum foil and incubated for 30 min with shaking at 250 rpm. Finally, 100 µL of stop solution was added to each well, and the side of the plate was tapped with fingertips until no blue was visible; the plate was immediately read at 450 nm. For a complete list of resources, see Table S1.

### 3.4. Data Acquisition and Analysis

Data were acquired using a synergy/H1 microplate reader. The OD values were then analyzed by Gen5 software. There were 36 replicates for both positives and negatives, and three replicates for each standard and archive samples. “No plasma” samples had six replicates. Mean OD, SD, and CV% were calculated for each sample by Gen5 software. The standard curve was constructed using the non-linear 4-parameter curve fit method. Titers were calculated based on the standard curve. The percent deviation of the highest and lowest single OD value from the mean OD value was calculated in Excel. For a complete list of resources, see Table S1.

## 4. Conclusions

We developed a more objective, quantitative, and high-throughput serological method to detect exposure to *R. parkeri* that can be optimized for other SFG rickettsiae. ELISA results are reported in titers that will be intuitive for clinicians accustomed to the interpretation of IFA data.

## Data Availability

The data presented in this study are available on request from the corresponding author.

## Supplementary Materials

The following are available online at https://www.mdpi.com/2076-0817/10/2/88/s1. Figure S1: Secondary antibody titration; Table S1: Complete list of reagents and resources; Table S2: Intra-plate reproducibility: Experiment 1; Table S3: Intra-plate reproducibility: Experiment 2; Table S4: Intra-plate reproducibility: Experiment 3; and Table S5: Inter-plate reproducibility.
