# Supplementary material for: Beyond the IFA: Revisiting the ELISA as a More Sensitive, Objective, and Quantitative Evaluation of Spotted Fever Group Rickettsia Exposure
Source: Pathogens. 2021 Jan 20;10(2):88. doi: 10.3390/pathogens10020088 (PMC7909427; doi:10.3390/pathogens10020088)
Supplement: Supplementary file 1 [file pathogens-10-00088-s001.pdf]

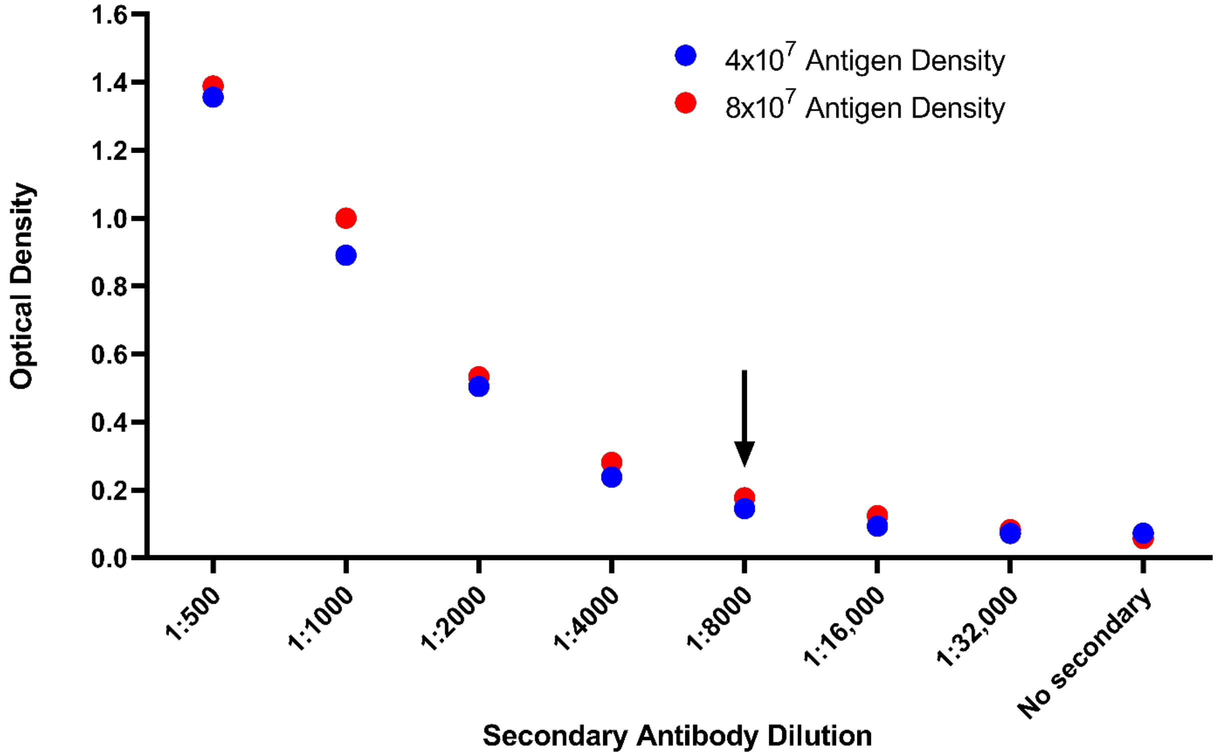

Figure S1. Secondary antibody titration. Initial titration of secondary antibody to determine the optimal concentration to be used in the assay. The graph demonstrates the optical density (OD) values for different secondary antibody serial dilutions from 500 to 32,000 for two different antigen densities: $4x10^7$ and $8x10^7$ rickettsiae per well. The arrow shows the inflection point of 1:8000, which we used in the assay until the optimal dilution (1:12,000) was determined later.

Table S1. Complete list of reagents and resources

REAGENT *or* RESOURCE	VENDOR	CATALOG NUMBER
Sample Collection		
EDTA microcontainers	BD	365974
Fetal Bovine Serum (FBS)	R&D Systems	S11510H
Tryptose Phosphate Broth	Sigma	T9157-100G
Antigen Preparation		
Minimum Essential Medium (MEM)	Gibco	11095-080
Fetal Bovine Serum (FBS)	R&D Systems	S11510H
60 mL syringes	BD	309653
25-gauge (1/2 or 5/8 in) needles	BD	305122
27-gauge (1/2 in) needles	BD	305109
18-gauge (3 in) needles	BD	305195
Cell Scraper (18 cm handle/ 1.8 cm blade)	Falcon	353085
3 µm Nuclepore® PC filter (in Swin-Lok™ 47mm holder)	Whatman	111112 (filter); 420400 (holder)
Ca+2 and Mg+2 free PBS, pH 7.4	Gibco	10-010-049
LIVE/DEAD™ BacLight™ Bacterial Viability Kit	Invitrogen	L7012
Petroff-Hausser counting chamber	Hausser Scientific	3900
Plating		
ELISA Strips (slightly hydrophilic)	Thermo Fisher Scientific	467120
5X ELISA Coating Buffer	Bio Rad	BUF030A
10x ELISA Wash Buffer	Bio Rad	BUF031A
Millipore Purified Water (0.1 µm filtered)	Millipore-Sigma	
ELISA Ultrablock	Bio Rad	BUF033A
Thermo Scientific™ Nunc™ Sealing Tape	Thermo Fisher Scientific	232698
Assay		
Goat anti-Guinea Pig IgG (H+L) Secondary Antibody, HRP	Thermo Fisher Scientific	A18775
TMB Substrate Solution	Thermo Fisher Scientific	N301
Stop Solution for TMB Substrate	Thermo Fisher Scientific	N600
Thermo Scientific™ Nunc™ Sealing Tape	Thermo Fisher Scientific	232698
10x ELISA Wash Buffer	Bio-Rad	BUF031A
Millipore water (0.1 micron filtered)	Millipore-Sigma	
Ca+2 and Mg+2 free PBS, pH 7.4	Gibco	10-010-049
Bovine Serum Albumin (BSA)	Sigma	A3059-100G
0.1-micron filters	PALL	4481
Protein LoBind Tubes	Eppendorf	022431064
Instruments and Software		
Gen5 (version 3.09)	BioTek	https://www.biotek.com/
Synergy/H1 microplate reader	BioTek	https://www.biotek.com/
Fisherbrand™ accuWash™ microplate washer	Thermo Fisher Scientific	https://www.fishersci.com/

Table S2. Intra-plate reproducibility: Experiment 1

Plate 1								
	**Mean OD**	**SD**	**CV%**	**High OD**	**High % deviation**	**Low OD**	**Low % deviation**	**Calculated titers**
**Positive control**	1.356	0.052	3.8	1.469	8.3	1.257	-7.3	601
**Negative control**	0.151	0.006	4.3	0.163	7.9	0.137	-9.3	38
**Archives**	2.536	0.078	3.1	2.612	3.0	2.457	-3.1	1215
**No plasma**	0.066	0.003	5.0	0.070	6.1	0.063	-4.5	OR
**Standard 1200**	2.475	0.064	2.6	2.545	2.8	2.419	-2.3	1200
**Standard 600**	1.356	0.021	1.6	1.380	1.8	1.341	-1.1	601
**Standard 300**	0.662	0.019	2.9	0.679	2.6	0.641	-3.2	297
**Standard 150**	0.351	0.014	4.1	0.366	4.3	0.338	-3.7	155
**Standard 75**	0.199	0.004	2.0	0.204	2.5	0.196	-1.5	72

First experiment displaying mean OD, SD, CV%, highest single OD (High OD), high % deviation (% deviation of highest single OD from mean OD), lowest single OD (Low OD), low % deviation (% deviation of lowest single OD from mean OD), and calculated titers (calculated from standard curve). *OR = outside range of the standard curve.

Table S3. Intra-plate reproducibility: Experiment 2

Plate 2								
	**Mean OD**	**SD**	**CV%**	**High OD**	**High % deviation**	**Low OD**	**Low % deviation**	**Calculated titers**
**Positive control**	1.409	0.054	3.9	1.518	7.7	1.288	-8.6	652
**Negative control**	0.149	0.006	3.9	0.161	8.1	0.139	-6.7	48
**Archives**	2.658	0.107	4.0	2.778	4.5	2.573	-3.2	OR
**No plasma**	0.064	0.001	1.3	0.066	3.1	0.064	0.0	OR
**Standard 1200**	2.438	0.052	2.1	2.498	2.5	2.401	-1.5	1200
**Standard 600**	1.304	0.040	3.0	1.344	3.1	1.265	-3.0	599
**Standard 300**	0.697	0.012	1.8	0.707	1.4	0.683	-2.0	302
**Standard 150**	0.368	0.014	3.8	0.384	4.3	0.357	-3.0	148
**Standard 75**	0.209	0.004	1.9	0.213	1.9	0.206	-1.4	76

Second experiment displaying mean OD, SD, CV%, highest single OD (High OD), high % deviation (% deviation of highest single OD from mean OD), lowest single OD (Low OD), low % deviation (% deviation of lowest single OD from mean OD), and calculated titers (calculated from standard curve). *OR = outside range of the standard curve.

Table S4. Intra-plate reproducibility: Experiment 3

Plate 3								
	**Mean OD**	**SD**	**CV%**	**High OD**	**High % deviation**	**Low OD**	**Low % deviation**	**Calculated titers**
**Positive control**	1.615	0.068	4.2	1.779	10.2	1.500	-7.1	591
**Negative control**	0.169	0.006	3.6	0.185	9.5	0.159	-5.9	25
**Archives**	2.875	0.105	3.6	2.996	4.2	2.807	-2.4	1168
**No plasma**	0.071	0.004	6.2	0.079	11.3	0.067	-5.6	OR
**Standard 1200**	2.930	0.148	5.0	3.055	4.3	2.767	-5.6	1164
**Standard 600**	1.644	0.019	1.2	1.661	1.0	1.624	-1.2	602
**Standard 300**	0.786	0.033	4.2	0.824	4.8	0.767	-2.4	295
**Standard 150**	0.425	0.010	2.3	0.433	1.9	0.415	-2.4	159
**Standard 75**	0.235	0.007	2.9	0.243	3.4	0.230	-2.1	69

Third experiment displaying mean OD, SD, CV%, highest single OD (High OD), high % deviation (% deviation of highest single OD from mean OD), lowest single OD (Low OD), low % deviation (% deviation of lowest single OD from mean OD), and calculated titers (calculated from standard curve). *OR = outside range of the standard curve.

Table S5. Inter-plate reproducibility

Inter-plate Reproducibility						
	**Exp 1 Mean OD**	**Exp 2 Mean OD**	**Exp 3 Mean OD**	**Inter-Plate Mean OD**	**SD**	**CV%**
**Positive control**	1.356	1.409	1.615	1.460	0.137	9.4
**Negative control**	0.151	0.149	0.169	0.156	0.011	7.1
**Archives**	2.536	2.658	2.875	2.690	0.172	6.4
**No plasma**	0.066	0.064	0.071	0.067	0.004	5.4
**Standard 1200**	2.475	2.438	2.930	2.614	0.274	10.5
**Standard 600**	1.356	1.304	1.644	1.435	0.183	12.8
**Standard 300**	0.662	0.697	0.786	0.715	0.064	8.9
**Standard 150**	0.351	0.368	0.425	0.381	0.039	10.2
**Standard 75**	0.199	0.209	0.235	0.214	0.019	8.7

Inter-plate reproducibility was determined by calculating inter-plate mean OD, SD, and CV% values.
